# The “coma sign”: An iatrogenic complication of neurological assessment

**Published:** 2019-01-05

**Authors:** Brandon Lucke-Wold, Christopher Robinson DO

**Affiliations:** 1 Department of Neurosurgery, University of Florida College of Medicine, Gainesville, Florida, United States; 2 Division of Neurocritical Care, University of Florida College of Medicine, Gainesville, Florida, United States

**Keywords:** Coma, Sternal Rub, Supraorbital Pressure, Neurological Assessment

Acute brain injury is a commonly encountered clinical problem that can make appropriate clinical assessment challenging. A key limitation in the neurological intensive care unit is how examiners assess a patient’s level of arousal or responsiveness. The vast majority of providers are trained to use either nail bed pressure or a sternal rub technique to achieve such responses. While these noxious stimuli do elicit findings in non-comatose patients, comatose patients may be exposed to unnecessary harm with these techniques. In fact, reports in the literature have shown that sternal rubbing causes iatrogenic complications including skin tears, abrasions, and ecchymosis.^1^ Such consequences should be considered during formal neurological assessment. Here, we present a case of repetitive noxious stimuli leading to diffuse sternal bruising, which the authors formally refer to as the “coma sign” (Figure 1).

**Figure 1:**
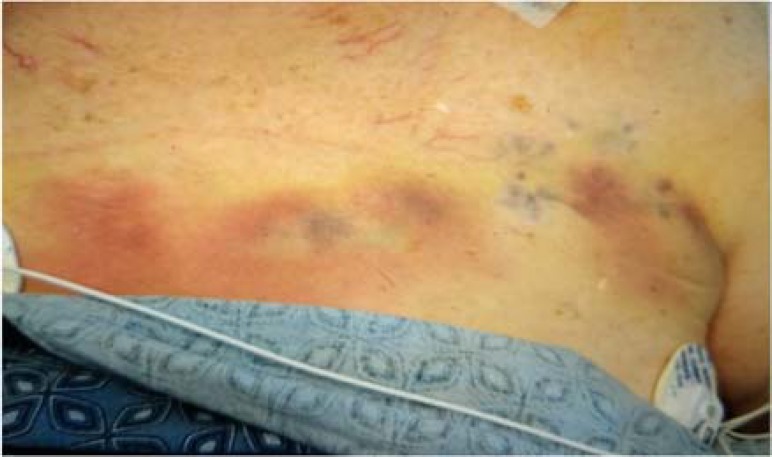
The “coma sign” from repetitive sternal rubs

Our patient was a 71-year-old woman who was admitted for subdural hematoma after striking her head on a curb. Upon arrival she had a Glasgow coma scale (GCS) score of 7, was intubated, and taken for emergent decompressive hemicraniectomy. Her postoperative course was complicated by refractory status epilepticus, which persisted for 2 weeks.

Care was ultimately transitioned to palliative measures and the patient expired shortly thereafter. Throughout the patient’s clinical course, as serial neurologic assessments were performed, evidence of a diffuse and progressive lesion appearing on the chest became apparent. Such lesion was identified as a consequence of repetitive sternal rubbing, and is what we refer to as the “coma sign.” Following recognition of such iatrogenic phenomena, alternative examination techniques including supraorbital pressure were recommended.

While frequent neurologic assessments are the backbone of determining patient status in the neurologic intensive care unit, patient safety should take priority. Sternal rub producing the “coma sign” is an archaic test. Supraorbital pressure in the supraorbital groove provides equally noxious stimuli for the patient but requires less applied force, and limits the potential harm to the patient (Figure 2). We argue that supraorbital pressure should be the go to test for assessing responsiveness in the neurologic intensive care unit, and should be supplemented with nail bed pressure if indicated to localize deficits. Supraorbital pressure also works more efficiently into the workflow of the neurologic exam as it can be assessed immediately before or after pupil checks. We are hopeful that increased awareness of this important topic can serve as the catalyst for instituting changes in how medical providers are trained to perform neurologic exams.

**Figure 2:**
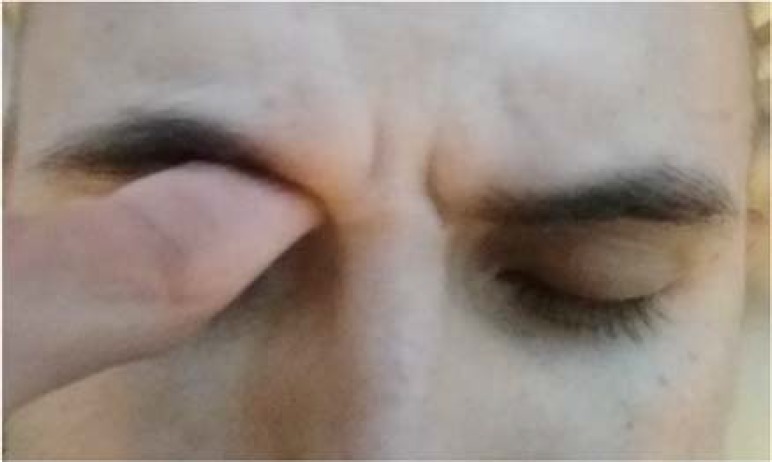
Supraorbital pressure providing sufficient noxious stimulation

## Notes:

**How to cite this article:** Lucke-Wold B, DO CR. The “coma sign”: An iatrogenic complication of neurological assessment. Iran J Neurol 2019; 18(1): 41-2.
